# Human and porcine aortic valve endothelial and interstitial cell isolation and characterization

**DOI:** 10.3389/fcvm.2023.1151028

**Published:** 2023-06-20

**Authors:** D. Nehl, P. R. Goody, K. Maus, A. Pfeifer, E. Aikawa, F. Bakthiary, S. Zimmer, G. Nickenig, F. Jansen, M. R. Hosen

**Affiliations:** ^1^ Heart Center Bonn, Molecular Cardiology, Department of Internal Medicine II, University Hospital Bonn, Bonn, Germany; ^2^ Institute of Pharmacology and Toxicology, University Hospital Bonn, Bonn, Germany; ^3^ Harvard Medical School, Brigham and Women's Hospital, Boston, MA, United States; ^4^ Heart Center Bonn, Department of Cardiac Surgery, University Hospital Bonn, Bonn, Germany

**Keywords:** aortic valve stenosis, valvular endothelial cells, valvular interstitial cells, endothelial-to-mesenchymal transition, calcification

## Abstract

**Background:**

Calcific aortic valve stenosis (AVS) is defined by pathological changes in the aortic valve (AV) and their predominant cell types: valvular interstitial (VICs) and endothelial cells (VECs). Understanding the cellular and molecular mechanisms of this disease is a prerequisite to identify potential pharmacological treatment strategies. In this study, we present a unique aortic valve cell isolation technique to acquire specific human and porcine cell populations and compared VICs and VECs of these species with each other for the first time.

**Methods:**

AV cells were isolated from tissue obtained from human patients undergoing surgical aortic valve replacement (SAVR) or from porcine hearts. Functional analysis and *in vitro* experiments revealed that endothelial-to-mesenchymal transition (EndMT) can be induced in hVECs, leading to a significant increase in mesenchymal markers. *In vitro* calcification experiments of VICs demonstrated pronounced expression of calcification markers and visible calcific deposits in Alizarin Red staining in both species after incubation with pro-calcific media.

**Results:**

Cells isolated from patient-derived AVs showed mesenchymal and endothelial-specific gene signatures (VIC and VEC, respectively). For instance, von Willebrand factor (*vWF*) and platelet endothelial adhesion molecule-1 (*PECAM1*) were upregulated in VECs, while the myofibroblastic markers alpha-smooth muscle actin (*α-SMA*) and vimentin (*VIM*) were downregulated in VECs compared to VICs. Analysis of cell function by migration revealed that VECs are more migratory than VICs. Induction of EndMT *in vitro* in VECs displayed increased expression of EndMT markers and decreased expression of endothelial markers, confirming their mesenchymal transdifferentiation ability. *In vitro* calcification of VICs revealed upregulation of alkaline phosphatase (*ALPL*), a hallmark of calcification. In addition, other calcification-related genes such as osteocalcin (*BGLAP*) and runt-related factor 2 (*RUNX2*) were upregulated. Alizarin red staining of calcified cells provided a further layer of confirmation that the isolated cells were VICs with osteoblastic differentiation capacity.

**Conclusion:**

This study aims to take a first step towards standardizing a reproducible isolation technique for specific human and porcine VEC and VIC populations. A comparison of human and porcine aortic valve cells demonstrated that porcine cells may serve as an alternative cellular model system in settings where human tissue is difficult to obtain.

## Graphical Abstract

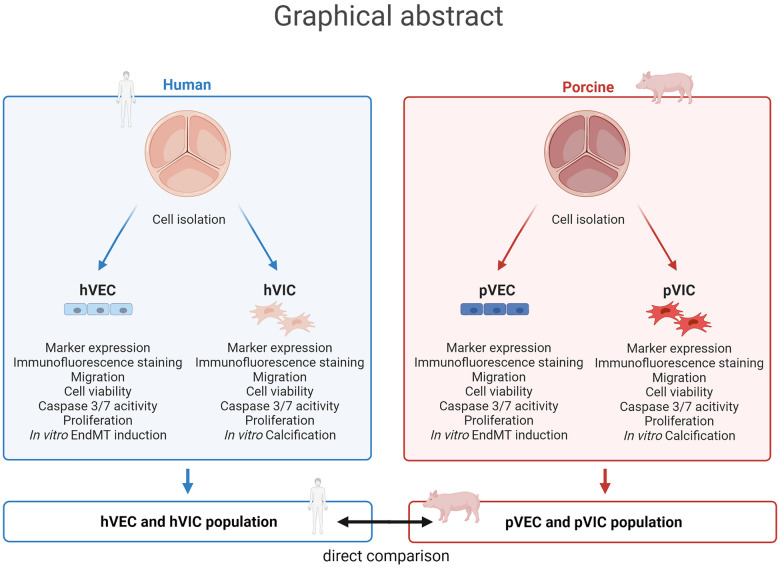

Aortic valve cell isolation: a comparative study of endothelial and interstitial cell isolation, and characterization with valvular tissue from porcine and humans. Human aortic valve endothelial cells (hVECs) and interstitial cells (hVICs) as well as porcine aortic valve endothelial cells (pVECs) and interstitial cells (pVICs) were isolated from human explants from patients undergoing surgical aortic valve replacement or porcine valvular tissue, respectively. To ensure specific cell populations, cells were characterized by marker expression analysis, immunofluorescence staining, and migration, cell viability, apoptosis, and proliferation assays. Additionally, endothelial-to-mesenchymal transition (EndMT) was induced *in vitro* in hVECs and pVECs and *in vitro* calcification of hVICs and pVICs. Finally, these results were compared from human cells to porcine cells and provide an alternative cellular model for aortic valve research.

## Introduction

Aortic valve stenosis (AVS) due to calcific aortic valve disease (CAVD) is one of the most common heart valve diseases worldwide, with a 2-year mortality of more than 50% when symptomatic (1–3). CAVD is characterized by fibrous thickening and calcification of the aortic valve cusps, resulting in a narrowing of the valve orifice. Originally described as a passive and degenerative disease, it is now well established that CAVD is a cell-mediated, actively regulated inflammatory disease (4–7). Currently, there are no approved medical therapies to prevent or reverse aortic valve calcification. The only treatment options are open heart surgery with aortic valve replacement (SAVR) or transcatheter aortic valve implantation (TAVR) (8). It is of upmost importance to better understand the mechanisms underlying this disease in order to identify feasible pharmaceutical and non-surgical/interventional therapies.

In healthy humans, the tricuspid aortic valve (AV) consists of three cusps covered on the aortic and ventricular sides by valvular endothelial cells (VECs). The interstitium of the valve is composed of three main layers with ubiquitously distributed valvular interstitial cells (VICs). During the pathogenesis of AVS, various stimuli, including shear stress and mechanical strain, induce VEC dysfunction, resulting in lipoprotein/phospholipid infiltration and oxidation (9–11). VECs lose their protective and regulatory properties and can transdifferentiate into myofibroblast-like VICs, a process termed endothelial-to-mesenchymal transition (EndMT) (12–14). Disruption of the endothelial layer also allows immune cell infiltration into the AV, which promotes the release of pro-inflammatory stimuli and drives osteogenic differentiation of VICs (9, 15). This osteogenic differentiation of VICs from a fibroblastic to an osteogenic phenotype induces the formation of calcium nodules, leading to AV calcification (9, 16). It is pivotal to further investigate these molecular and cellular mechanisms of AV cell populations involved in CAVD. However, isolation and cultivation of VECs and VICs is technically and methodologically highly demanding. Here, we provide a novel method for AV cell isolation that can be applied in all cell culture laboratories.

A variety of organisms can be used for such cell culture experiments, including humans and pigs (17–20). In general, human material is advantageous over animal material when studying potential therapeutic targets involved in human disease pathogenesis. While tissue from patients with CAVD can be used for genetic screening, cells from non-stenotic and healthy patients are the most useful and authentic platform to study disease progression (e.g., induction of osteoblastic differentiation and EndMT). Calcified cusps of diseased human AVs can be obtained from patients undergoing AV replacement surgery for severe AVS. Control tissues are more difficult to obtain because non-calcified aortic valves are less frequently explanted (e.g., in the setting of aortic root or ascending aortic dilatation/aneurysm, when aortic regurgitation occurs as a result). It is therefore important to investigate whether other organisms genetically comparable to humans can be used as tissue donors for cell isolation. It is undoubtedly challenging to obtain high-quality control samples from healthy or non-stenotic AVs. In addition to the most obvious problems, individual patients naturally exhibit a high variability in age, sex, congenital, pre-existing, and concomitant diseases (21). Thus, the degree of calcification patterns varies greatly between patients and between sexes. Because of these difficulties, the most commonly used AV tissues for cell isolation are obtained from large animals that are genetically similar to humans, such as pigs (22–24). An advantage of porcine tissue over human material is that healthy tissue is more readily available from abattoirs or animal research facilities. The hearts and AVs are healthy and unaffected by other diseases, and the cells can therefore be isolated and expanded more efficiently *in vitro*. The size and the substantial amount of tissue in porcine AVs makes it easier to study the mechanical properties of the cusps or to isolate cells. Moreover, the whole heart can be used for research, whereas in humans only one valve is usually explanted (25).

Although porcine valves appear to be anatomically and genetically similar to human valves, there may be differences in specific molecular pathways. To date, no direct comparison of human and porcine VECs and VICs has been performed (26). In this study, we undertook a comprehensive comparison of porcine and human AV cells to identify a more readily available alternative to human material for cell culture experiments. Cells were isolated in a cell population-specific manner and further characterized. Cell culture experiments, such as *in vitro* EndMT and calcification induction, were performed on human and porcine cells to analyze and compare their response to these external stimuli.

## Materials and methods

### Human and porcine aortic valve cell isolation

Human AV samples were obtained from patients undergoing AV replacement for aortic regurgitation due to aortic root or ascending aortic dilatation at the Heart Center Bonn, University Hospital Bonn, Germany. The study protocol was approved by the Ethics Committee of the University Hospital Bonn (approval number AZ 078/17). All patients signed an informed consent and the study was performed in accordance with the Declaration of Helsinki and the International Conference on Harmonization of Good Clinical Practice (27). After explantation, AV cusps were placed in 1× PBS on ice and immediately transported to the laboratory for cell isolation. Porcine AVs were obtained from the abbatoir Landschalchterei Schmitz in Euskirchen, Germany. Freshly slaughtered pigs served as donors, and the hearts were directly rinsed with NaCl and placed in 500 ml cold 1 × PBS on ice and supplemented with 2,500 U heparin-sodium (B. Brown). Three AV cusps were excised from the aortic root. Immediately after explantation, human aortic valve endothelial cells (hVECs), human aortic valve interstitial cells (hVICs), porcine aortic valve endothelial cells (pVECs), and porcine aortic valve interstitial cells (pVICs) were isolated from the three cusps starting with an incubation in 600 U/ml collagenase II in VIC primary medium (DMEM (Thermo Fisher Scientific, 21885108) supplemented with 10% FBS (Gibco, #A3160802), 1% penicillin/ streptomycin (pen/ strep) (Merck, #516106), and 44 mM NaHCO_3_ (AppliChem, #A1353)) for 10 min at 37 °C with gentle agitation. VECs were gently scraped from the cusp surface into a dish containing VEC medium [EGM™-2 MV Microvascular Endothelial Cell Growth Medium-2 BulletKit™ (Lonza, #C-3202)] using a sterile No.10 scalpel (Feather, #02.001.30.010) positioned at a 45° angle to the cusp at a slow but steady speed. Close-up of a cusp after scratch showed a roughening at the surface (Supplementary Figure S1A). Cells were washed twice with VEC medium by centrifugation at 1,000 rpm for 15 min and filtered through a 100 µm cell strainer. To ensure a specific VEC suspension, cells were magnetically labeled with CD105 magnetic beads (Miltenyi Biotech, #130-051-201) and a MACS separation was performed according to the manufacturer's protocol. VECs were seeded on fibronectin-coated (Sigma-Aldrich, #F0895) (5 µg/cm^2^) T25 flasks. To isolate VICs, the remaining valve tissue was minced and further digested with 600 U/ml collagenase II in VIC primary medium for 20 h at 37° C with gentle agitation. The cell suspension was then filtered (100 µm) and centrifuged twice at 1,000 rpm for 15 min. VICs were seeded on collagen-coated (5 µg/cm^2^) T75 flasks.

### Cell culture

All cells were detached using a Lonza detachment kit (Lonza, #CC-5034) and cultured in sterile incubators at 37°C, 5% (v/v) CO_2_, and 95% humidity. hVECs and pVECs were cultured in EGM™-2 MV Microvascular Endothelial Cell Growth Medium-2 BulletKit™ (Lonza, #CC-3202). hVICs and pVICs were cultured in VIC medium, consisting of DMEM (Thermo Fisher Scientific, 21885108), 10% FBS (Gibco, #A3160802), and 1% penicillin/streptomycin (pen/strep) (Merck, #516106). All experiments were performed in the fifth or sixth passage at 70% confluence on 12-, 24-, or 96-well plates.

### Migration assay

Cell migration was analyzed by the scratch wound healing assay (28) on a 24-well plate. hVECs and pVECs were incubated with starvation medium, VEC medium without FBS, for 18 h and then scratched with a sterile 200 µl pipet tip. The medium was changed to normal VEC medium and scratch wound closure was documented every 2 h by photographing with a Zeiss Axiovert 200 M microscope. The remaining cell-free area under the scratch was analyzed using Zen Lite software (Zeiss).

### Cell viability, apoptosis, and proliferation assay

Cells were treated with 100 µM H_2_O_2_ for 24 h. Cell viability was then analyzed using alamarBlue™ cell viability reagent (Thermo Fisher Scientific, # DAL1025), which was added to the cell medium according to the manufacturer's instructions, and absorbance was measured using the Microplate Reader Infinite® M Plex (Tecan). Apoptosis was measured using the Caspase-Glo® 3/7 Assay System (Promega, #G8091), according to the manufacturer's protocol using the Microplate Reader Infinite®M Plex (Tecan) to measure luminescence. Proliferation capacity was analyzed by BrdU staining. Cells were treated with BrdU (BD Pharmigen™, #51-2420KC) for 24 h, washed with 1× PBS and fixed with 4% PFA. After three more washing steps, cells were incubated with 1% Triton X-100 in 1× PBS for 3 × 5 min, followed by incubation with 1 N HCl on ice for 10 min. After changing the medium to 2 N HCl and incubating for 10 min at RT, the cells were incubated for another 20 min at 37°C. Cells were washed with 0.1 M di-sodium tetraborate for 12 min, followed by 3 washing steps with 1% Triton X-100 in 1× PBS for 5 min. Cells were blocked with 1% Triton X-100 in 1× PBS, supplemented with 1 M glycine and 5% normal goat serum for 1 h. Incubation with the primary rat monoclonal antibody anti-BrdU (Abcam, #ab6326) was performed overnight in 1% bovine serum albumin (BSA) in 1× PBS, followed by three washing steps with 1% Triton X-100 and the incubation with the secondary antibody donkey anti-rat Cy3 (Dianova, #115-165-146) for one hour. Finally, after three further washing steps with 1% Triton X-100, cells were embedded in antifade mounting medium containing DAPI (Vectashield, #H-1200-10) and fluorescence microscopy was performed using an Axio Observer inverted microscope (Carl Zeiss, Jena, Germany).

### Doubling time calculation

The doubling time/cell lifetime in cell culture was calculated using the following formula: Doubling time = (time × log2)/log (final cell number/initial cell number). The time is the elapsed time, the final cell number is the number of cells at the end of the period, and the initial cell number is the number of cells at the beginning of the period.

### *In vitro* endMT induction

*In vitro* endothelial-to-mesenchymal transition (EndMT) was induced in hVECs and pVECs in a 12-well plate using EGM™-2 MV Microvascular Endothelial Cell Growth Medium-2 BulletKit™ (Lonza, #CC-3202), without VEGF, GA-1000, and hydrocortisone, but supplemented with either 30 ng/ml tumor necrosis factor alpha (TNF*α*; R&D Systems, #210-TA-020), 1–10 ng/ml tumor necrosis factor beta 1 (TGFβ1; R&D Systems, #240-B-10)) or TGF*β*1 in combination with 1 ng/ml interleukin 1 beta (IL1β; R&D Systems, #201-LB-010) for 7 days. EndMT induction was verified by gene expression analysis using RT-qPCR.

### *In vitro* calcification model

For *in vitro* calcification, hVICs and pVICs were seeded on 12-well plates and cultured with DMEM (Thermo Fisher Scientific, #21885108), 5% FBS (Gibco, #A3160802), and 1% penicillin/streptomycin (pen/strep) (Merck, #516106), osteogenic medium (OM), or pro-calcifying medium (PCM). OM consisted of DMEM with 5% FBS, 1% pen/strep, 10 nmol/L β-glycerophosphate (Sigma-Aldrich, #G9422), 10 mmol/L dexamethasone (Sigma-Aldrich, #D4902), and 50 µg/ml L-ascorbic acid (Carl Roth, #3525.2). PCM consisted of DMEM with 5% FBS, 1% pen/strep, 2 mmol/L sodium dihydrogen phosphate (NaH_2_PO_4_; Merck, #71507), and 50 µg/ml L-ascorbic acid (Carl Roth, #3525.2). Cells were incubated for 7 days to analyze gene expression by RT-qPCR and for 21 days for calcium deposition staining with 2% alizarin red (Sigma-Aldrich, #A5533). Cells were fixed with 4% formaldehyde for 15 min and washed twice with distilled water. Calcium nodules were stained with Alizarin Red for 15 min at room temperature, followed by two additionally washed. Staining was quantified by incubating cells with 3.58% hexadecylpyridinium chloride monohydrate (CPC) dissolved in ddH_2_O for 1 h. The absorbance of 100 µl supernatant was measured at 550 nm using an Infinite® M Plex microplate reader (Tecan).

### RNA isolation and real time PCR-based gene expression profiling

RNA from cultured VICs and VECs was extracted using TRIzol reagent (Thermo Fisher Scientific, #15596018), and subsequent cDNA synthesis was performed using High-Capacity cDNA Reverse Transcription Kit (Thermo Fisher Scientific, # 43-688-13), both according to the manufacturer's instructions. RT-PCR-based gene expression profiling was performed using TaqMan™ Gene Expression Master Mix (Thermo Fisher Scientific, #4370074) and the following human TaqMan™ primers (Thermo Fisher Scientific): *GAPDH* (Hs02758991_g1), *α-SMA* (Hs00426835_g1), *vWF* (Hs01109446_m1), *PECAM1* (Hs01065279_m1), *VIM* (Hs00418522_m1), *CD105* (Hs00164438_m1), *NOS3* (Hs01574665_m1), *CDH2* (Hs00983056_m1), *VCAM1* (Hs00365486_m1), *SNAI2* (Hs00161904_m1), *ALPL* (Hs01029144_m1), *BGLAP* (Hs01587814_g1) and *RUNX2* (Hs01047973_m1). The following porcine TaqMan™ primers were used: *GAPDH* (Ss03375629_u1), *α-SMA* (Ss04245588_m1), *vWF* (Ss04322692_m1), *PECAM1* (Ss04322692_m1), *VIM* (Ss04330801_gH), *CD105* (Ss03391353_m1), *NOS3* (Ss03383840_u1), *CDH2* (Ss06911356_m1), *VCAM1* (Ss03390912_m1), *ALPL* (Ss06879568_m1), *BMP2* (Ss03373798_g1), *BGLAP* (Ss03373655_s1), and *SPP1* (Ss03391321_m1). *ΔΔ*CT values were calculated using Microsoft Excel (Microsoft Corp, Redmond, WA, USA) normalized to *GAPDH,* and significance was tested and visualized using GraphPad Prism (GraphPad Software, Inc., San Diego, CA, USA).

### Immunofluorescence staining

Cells were fixed with 4% formaldehyde for 30 min in 24-well plates with glass coverslips. After three washes with 1× PBS, cells were permeabilized with 0.25% Triton X-100 in PBS for 10 min and then washed three more times with 1× PBS. Cells were blocked with 1% BSA-glycine in PBS with 0.1% Tween for 30 min and then incubated with the primary antibodies for 2 h on a shaker. For hVECs and hVICs, alpha smooth muscle actin (*α*-SMA) (Abcam, #ab7817), vimentin (VIM) (Abcam, #8978), von Willebrand factor (vWF) (Abcam, #ab6994), and platelet adhesion molecule-1 (PECAM1) (Abcam, #28364) were used. For pVECs and pVICs, the vWF antibody was replaced with vWF (Bio-Rad, #AHP062T). Cells were washed three times with 1× PBS followed by incubation with the secondary antibody on a shaker for 1 h: goat IgG anti-mouse IgG Cy3 (Dianova, #115-165-146), goat IgG anti-rabbit IgG Cy2 (Dianova, #111-225-144), or donkey IgG anti-sheep IgG Cy2 (Dianova, #712-225-147). Again, cells were washed three times with 1× PBS and embedded in Vectashield® Antifade Mounting Medium with DAPI (Vectashield, #H-1200-10). Fluorescence microscopy was performed using an Axio Observer inverted microscope (Carl Zeiss, Jena, Germany).

### Statistical analysis

Data analysis was performed using Microsoft Excel (Microsoft Corp, Redmond, WA, USA). Data are presented as mean ± SEM. Unpaired and 2-tailed Student's *t*-test was performed with Prism 9 (GraphPad Software, Inc., San Diego, CA, USA).

## Results

### Cell isolation leads to specific hVEC and hVIC populations

**Figure 1:**
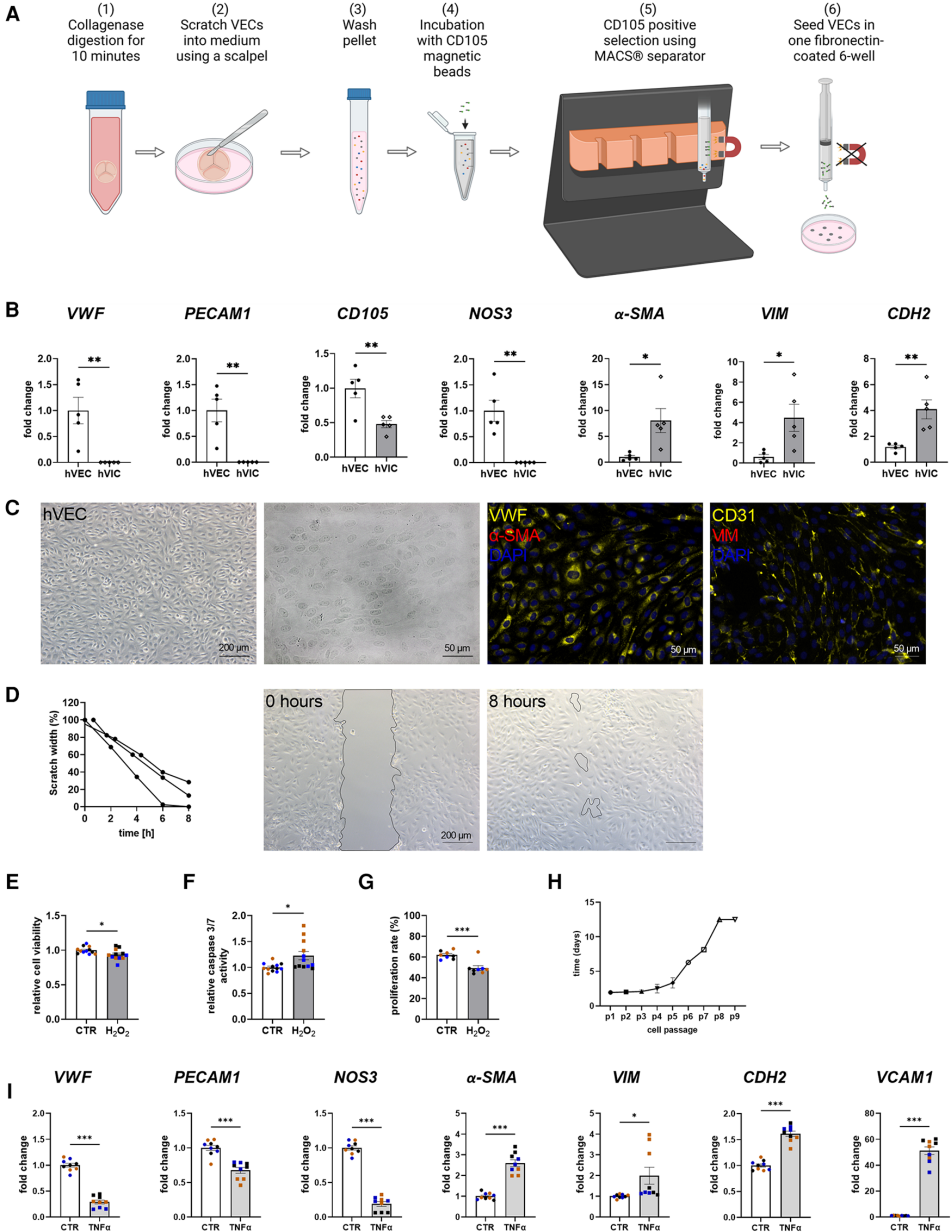
Human valvular endothelial cell (hVEC) isolation and characterization. (**A**) Workflow of VEC isolation: (1) Explanted aortic valve cusps were incubated for 10 min in collagenase type II solution. (2) VECs were carefully scraped with a scalpel into a dish filled with VEC medium, which (3) was transferred into a 15 ml tube and washed twice for 15 min at 1,000 rpm at room temperature. (4) Cells were incubated with CD105 magnetic beads for 15 min, and (5) the CD105 positive selection was carried out with MACS® separator, according to the manufacturer's protocol. (6) The specific VEC population was seeded into one well of a fibronectin-coated 6-well plate. (**B**) Gene expression of isolated hVECs and human valvular interstitial cells (hVICs) showed a positive expression of the endothelial markers von Willebrand factor (*vWF*), platelet adhesion molecule 1 (*PECAM1*), endoglin (*CD105*), and nitric oxide synthase 3 (*NOS3*) in hVECs. (**C**) Brightfield and immunofluorescence images of VECs positive for vWF and CD31 and negative for alpha smooth muscle actin (α-SMA) and vimentin (VIM). (**D**) Migration analysis by scratch wound healing assay resulted in a scratch width of 15% after 8 h. Treatment of hVECs with H_2_O_2_ for 24 h resulted in decreased (**E**) cell viability, increased (**F**) caspase 3/7 activity, and decreased (**G**) cell proliferation. (**H**) Life span of hVECs in cell culture at different passages (p). (**I**) Inducing EndMT by incubating VECs with tumor necrosis factor alpha (TNFα) for 7 days. TNFα led to an upregulation of *α*-*SMA, VIM*, cadherin 2 (*CDH2*) and vascular adhesion molecule 1 (*VCAM1*) and a downregulation of *PECAM1, vWF* and NOS3. (**B**) *n* = 5 donors, (**D**) *n* = 3 donors, (**E–I**) *n* = 3 donors with technical replicates indicated by one color per donor, *P < 0.05, **P < 0.01, ***P < 0.001, analyzed by Student *t*-test, 2-tailed, unpaired, (**A**) created with BioRender.com.

MACS-sorted hVECs and isolated hVICs (Figure 1A) were characterized by RT-qPCR to quantify cell-specific markers. Our quantification revealed an increased expression of endothelial markers, such as von Willebrand factor (*vWF*), platelet endothelial adhesion molecule-1 (*PECAM1*), endoglin (*CD105*), and nitric oxide synthase 3 (*NOS3*) in hVECs compared to hVICs. Myofibroblastic markers, alpha smooth muscle actin (*α-SMA*), vimentin (*VIM*), and cadherin 2 (*CDH2*) were less expressed (Figure 1B). Brightfield pictures of hVECs displayed typical endothelial morphology (Figure 1C). Immunofluorescence staining demonstrated an increase in intracellularly localized vWF and an expression of the endothelial marker CD31 whereas no expression of *α*-SMA and VIM was observed, further providing evidence that the isolated cells were hVEC (Figure 1C). Analysis of hVEC migration capacity by scratch wound closure revealed only 15% scratch width after 8 h (Figure 1D), suggesting that these cells maintain endothelial migration capacity, further confirming a specific hVEC population. While caspase 3/7 activity increased, cell viability and proliferation rate decreased after apoptosis induction with H_2_O_2_ (Figures 1E–G). Analysis of hVECs lifespan revealed a consistent doubling rate up to passage 5 (Figure 1H). To further assess if hVECs were able to undergo EndMT *in vitro*, cells were incubated with EndMT-inducing stimulants. First, cells were incubated with 1–10 ng/ml TGFβ1 for 7 days, which showed diffuse gene expression (Supplementary Figure S2A). The endothelial maker *vWF* was downregulated, *PECAM1* and *CDH5* were upregulated. The mesenchymal maker *a-SMA* was upregulated, but *SNAI2* and *VIM* were not. Therefore, 2 additional conditions were tested, namely 5 ng TGF*β*1 + 1 ng/ml IL1β and 30 ng/ml TNF*α*. Treatment with TGFβ1 + IL1β also did not show consistent expression of endothelial and mesenchymal markers (Supplementary Figure S2B). TNFα treated hVECs displayed increased expression of mesenchymal markers, such as *α-SMA*, *VIM, CDH2*, and vascular adhesion molecule 1 (*VCAM1*), and decreased expression of endothelial markers, namely *vWF, PECAM1*, and *NOS3* (Figure 1I), demonstrating the mesenchymal transdifferentiation capacity of hVECs upon TNF*α* incubation. These data suggest that elevated mesenchymal marker expression represents an effective EndMT induction.

**Figure 2:**
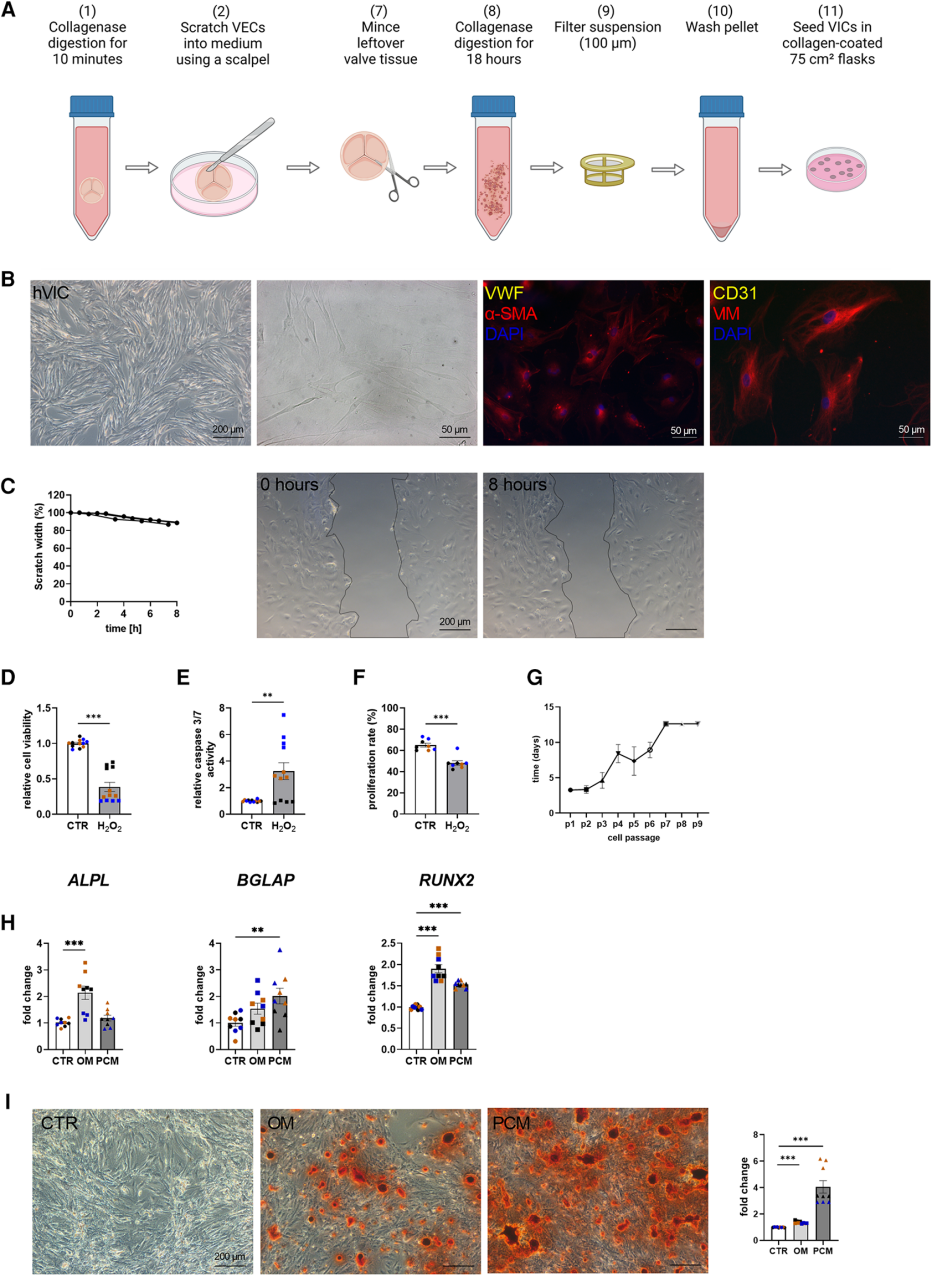
Human valvular interstitial cell (hVIC) isolation and characterization. (**A**) Workflow of VIC isolation: (7) the remaining aortic valve tissue after VEC removal (2) was minced into small pieces and (8) further digested in collagenase solution for 18 h. (9) Cell suspension was filtered (100 µm nylon mesh filter) and (10) the pellet was washed twice. (11) VICs were seeded into a collagen-coated 75 cm² flask. (**B**) Brightfield images and immunofluorescence staining of hVICs showing positive staining for alpha smooth muscle actin (α-SMA) and vimentin (VIM). (**C**) Migration analysis by scratch wound healing assay revealed in scratch width of 87% after 8 h. hVICs treated with 100 µM H_2_O_2_ for 24 h showed reduced (**D**) cell viability, increased (**E**) caspase 3/7 activity, and decreased (**F**) cell proliferation. (**G**) Cell culture lifespan of hVICs at different passages (p). (**H**) Gene expression analysis of hVICs incubated with osteogenic medium (OM) or pro-calcifying medium (PCM) for 7 days to induce *in vitro* calcification. QPCR analysis of alkaline phosphatase (*ALPL*) expression showed elevated expression in OM, but not in PCM. Osteocalcin (*BGLAP*) and bone morphogenic protein 2 (*BMP2*) expression was upregulated in OM and PCM, compared to control medium (CTR). (**I**) Alizarin red staining and quantification displayed increased calcium nodule formation in OM and PCM. (**C**) *n* = 3 donors, (**D–I**) *n* = 3 donors with technical replicates indicated by one color per donor, **P < 0.01, ***P < 0.001, analyzed by Student *t*-test, 2-tailed, unpaired, (**A**) created with BioRender.com.

Baseline characteristics indicated that comorbidities influencing the prognosis after SAVR [e.g., pulmonary disease and coronary artery disease (CAD)] were similar between the groups [CAVD vs. no CAVD]. Of note, other cardiovascular risk factors such as type-II diabetes (Type II DM), body mass index (BMI), dyslipidemia, smoking history, as well as creatinine levels, were noted to be not elevated in patients with CAVD (Table 1). Patient-derived hVICs were isolated (Figure 2A) and characterized by RT-qPCR to quantify interstitial marker gene expression. Whereas *α-SMA*, *VIM*, and *CDH2* expression were significantly upregulated, *vWF*, *PECAM1, CD105*, and *NOS3* showed decreased expression values compared to hVECs (Figure 1B). Brightfield images of hVECs showed a typical elongated mesenchymal cell shape (Figure 2B). Immunofluorescence staining of hVICs revealed a positive staining for intracellularly distributed *α*-SMA and VIM, but no expression of vWF and CD31 was detected (Figure 2B). To analyze the migration properties of hVICs, a scratch wound assay was performed, and the closure was observed for 8 h. The remaining scratch area was 87% after 8 h (Figure 2C). H_2_O_2_ treatment of hVICs revealed a decreased cell viability and proliferation rate and an increased caspase 3/7 activity (Figures 2D–F). The doubling rate of pVICs remained stable until passage 3, was prolonged at passage 4, and remained at this doubling time until passage 6 (Figure 2G). *In vitro* calcification, by incubating hVICs with OM or PCM for 7 days, revealed an overexpression of alkaline phosphatase (*ALPL*), a hallmark of calcification, in OM but not in PCM (Figure 2H). In addition, other calcification-related genes such as osteocalcin (*BGLAP*) and runt-related factor 2 (*RUNX2*) were upregulated in both conditions. Alizarin red staining and the staining quantification of calcified cells provided a further layer of confirmation that the isolated cells were VICs with osteoblastic differentiation capacity (Figure 2I). These data also confirmed that *in vitro* calcification of isolated hVICs was successfully induced.

**Table 1:** Baseline characteristics of the patients used for cell isolation.

	CAVD	no CAVD
Total population	5	5
**Clinical Parameters:**		
Male	3	5
Age (mean)	65.5 ± 8.4	74 ± 5
NYHA levels	2.6 ± 0.5	2.5 ± 0.5
CAD	3 (50)	5 (83.3)
Hypertension	4 (66.7)	6 (100)
AVA in cm^2^	0,74 ± 0.19	3.98 ± 1.3
Vmax in m/s	4.4 ± 0.31	1.9 ± 0.37
p_mean_ in mmHg	47 ± 12.8	N.D.
LV function (mean)	55.7 ± 3.88	52.4 ± 14.6
Mild-Severe Mitral regurgitation	2 (30)	3 (50)
**Cardiovascular risk factors:**		
Type II DM	2 (30)	0 (0)
BMI (mean)	25.4 ± 2.3	25.8 ± 3.2
Dyslipidemia	4 (66.7)	5 (83.3)
Smoker	1 (16.7)	1 (16.7)
Creatinine in mg/dl (mean)	0.86 ± 2.3	1.07 ± 0.55

Baseline demographic, and echocardiographic parameters of the validation study population. CAVD, calcified aortic valve disease; NYHA, New York heart association; CAD, coronary artery disease; Vmax, velocity maximum; Pmean, mean pressure; Type II DM, Type 2 diabetes mellitus; BMI, body mass index; LV, left ventricle; AVA, aortic valve area.

### Porcine aortic valve cell isolation revealed specific VEC and VIC cell populations

**Figure 3:**
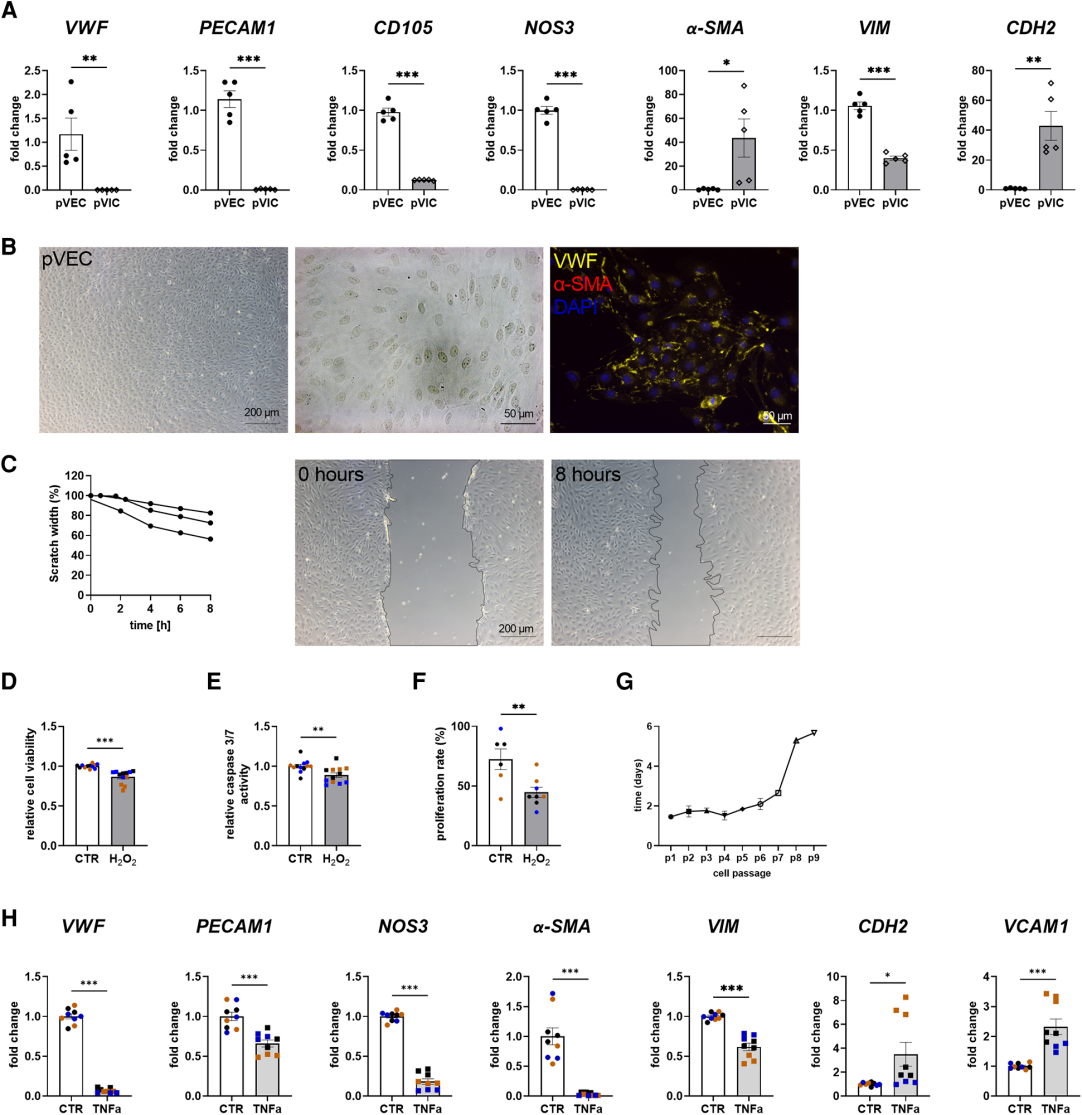
Porcine valvular endothelial cell (pVECs) isolation and characterization. (**A**) Gene expression analysis of pVECs revealed a significant upregulation of von Willebrand factor (*vWF*), platelet adhesion molecule 1 (*PECAM1*), endoglin (*CD105*), nitric oxide synthase 3 (*NOS3*), and vimentin (*VIM*) and a downregulation of alpha smooth muscle actin (*α-SMA*) and cadherin 2 (*CDH2*) compared to porcine valvular interstitial cells (pVICs). (**B**) Brightfield images and immunofluorescence staining of pVECs showing a positive signal for vWF and an absent signal for α-SMA. (**C**) Analysis of migration properties by scratch wound healing assay revealed a scratch closure to 70%. pVECs treated with 100 µM H_2_O_2_ showed a decrease in (**D**) cell viability, (**E**) caspase 3/7 activity, and (**F**) cell proliferation. (**G**) Life span of pVECs in cell culture at different passages (p). (**H**) *In vitro* EndMT induction by TNFα for 7 days. TNFα displayed downregulated endothelial marker expression, (*PECAM1, vWF, NOS3*) and mesenchymal markers, (*α-SMA, VIM, CDH2*). *CDH2* and vascular cell adhesion molecule 1 (*VCAM1*) was upregulated. (**A**) *n* = 5 donors, (**C**) *n* = 3 donors, (**D–H**) *n* = 3 donors with technical replicates indicated by one color per donor, *P < 0.05, **P < 0.01, ***P < 0.001, analyzed by Student *t*-test, 2-tailed, unpaired, (a) created with BioRender.com.

To assess whether porcine cells can be used alternatively for cell culture experiments instead of difficult-to-obtain human cells, porcine VECs (pVECs) and porcine VICs (pVICs) were characterized. Cells were isolated using the same protocol as for human cells (Figure 1A). Analysis of characteristic marker gene expression in isolated pVECs revealed elevated levels of *vWF*, *PECAM1*, *CD105*, and *NOS3* levels and decreased levels of *α-SMA* and *CDH2*, compared to pVICs. However, *VIM* was also upregulated in pVECs (Figure 3A). Brightfield images of pVECs showed a typical endothelial cell shape (Figure 3B). In immunofluorescence staining, positive staining for vWF was observed, but no *α*-SMA signal was detectable in pVECs (Figure 3B). To assess the migration behavior of hVECs, a scratch wound assay was performed, which revealed a scratch closure to 70% after 8 h (Figure 3C). The cell viability, caspase 3/7 activity, and proliferation rate after H_2_O_2_ treatment were downregulated in all three assays (Figures 3D–F). The lifespan of pVECs remained stable up to passage 7 (Figure 3G). These data provided evidence that pVECs displayed an endothelium-specific phenotype. However, *in vitro* EndMT induction with TNF*α* (Figure 3H) or TGFβ1 + IL1β (Supplementary Figure S2C) demonstrated a decrease in EndMT markers, indicating that the cells had not undergone efficient transdifferentiation.

**Figure 4:**
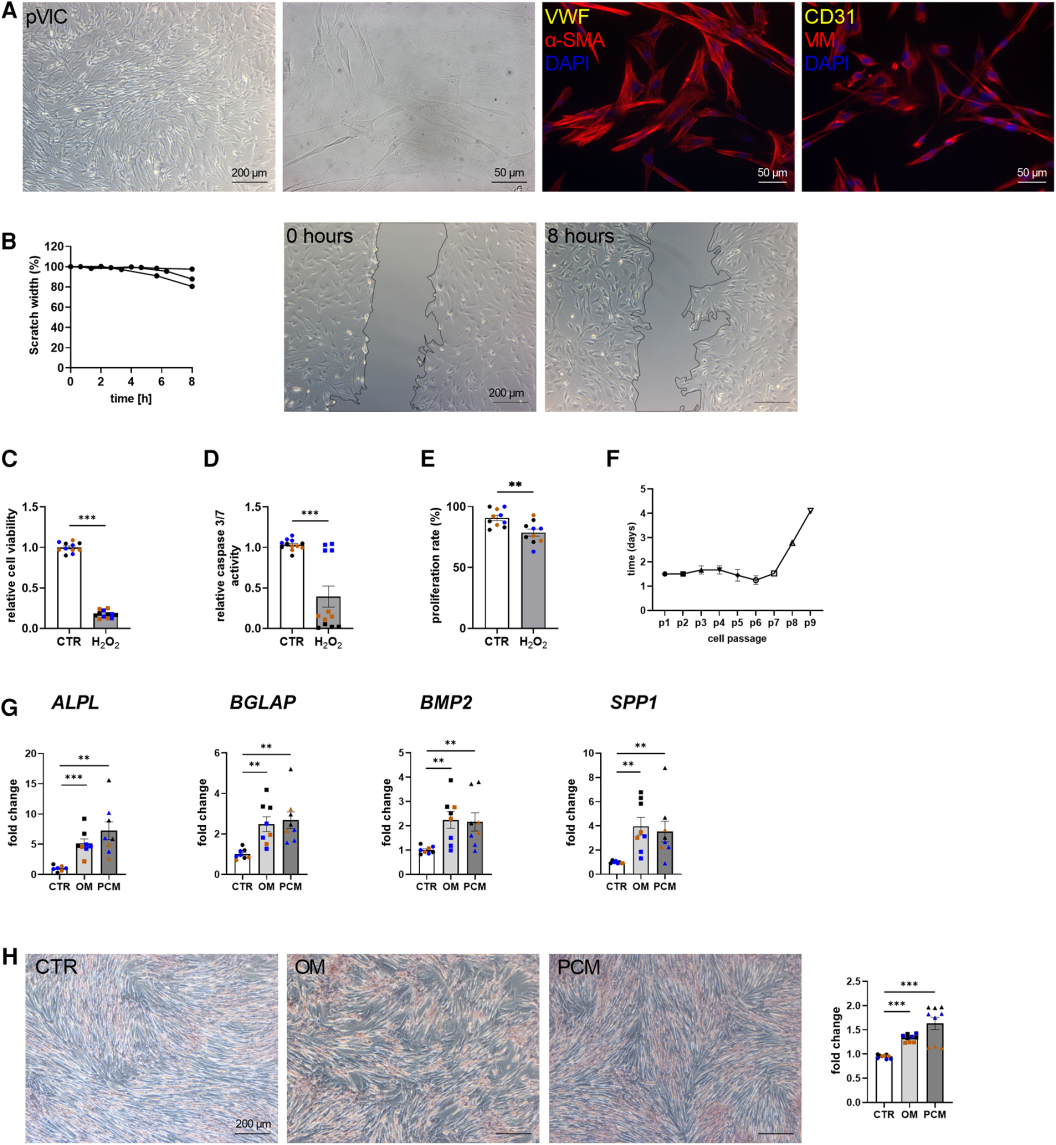
Porcine valvular interstitial cell (pVICs) isolation and characterization. (**A**) Brightfield and immunofluorescence images of pVICs displaying a positive staining for a-SMA and VIM and a negative signal for CD31 and vWF. (**B**) Migration assays revealed a scratch closure to 87% after 8 h. pVICs treated with 100 µM H_2_O_2_ for 24 h showed a decrease in (**C**) cell viability, (**D**) caspase 3/7 activity, and (**E**) cell proliferation. (**F**) Life span of pVICs in cell culture at different passages (p). (**G**) Gene expression analysis of pVICs treated with osteogenic medium (OM) or pro-calcifying medium (PCM) for 7 days to induce *in vitro* calcification. Calcification markers, such as alkaline phosphatase (*ALPL*), bone morphogenic protein 2 (*BMP2*), osteocalcin (*BGLAP*), and osteopontin (*SPP1*) were significantly upregulated in OM and PCM. (**H**) Alizarin Red staining and the quantification showed pronounced calcific nodules in OM and PCM, compared with control medium (CTR). (**B**) *n* = 3 donors, (**C–H**) *n* = 3 donors with technical replicates indicated by one color per donor, **P < 0.01, ***P < 0.001, analyzed by Student *t*-test, 2-tailed, unpaired, (**A**) created with BioRender.com.

To characterize the isolated pVICs, using the same protocol as for human cells (Figure 2A), characteristic interstitial cell markers were analyzed, showing an upregulation of *α-SMA* and *CDH2* in pVICs compared to pVECs (Figure 3A). The endothelial markers, *vWF*, *PECAM1 CD105, NOS3,* but also *VIM* was decreased. Brightfield images of pVICs showed a typical mesenchymal cell shape (Figure 4A). Immunofluorescence staining displayed a positive signal for *α*-SMA and VIM, while vWF and CD31 were undetectable (Figure 4A). Scratch-wound based functional assays revealed a scratch width after 8 h of 87% (Figure 4B). These data suggest a VIC-specific phenotype of the isolated cells. When pVICs were treated with H_2_O_2_ for 24 h, cells displayed reduced cell viability, caspase 3/7 activity and proliferation rate (Figures 4C–E). Analysis of the doubling time of pVICs showed a stable doubling time up to passage 7 (Figure 4F). To assess whether calcification can be induced in pVICs *in vitro*, cells were incubated with OM or PCM for 7 days, which showed a significant upregulation of calcification-related genes, namely *ALPL*, *BMP2*, *BGLAP*, and osteopontin *(SPP1*) in OM and PCM (Figure 4G). Additionally, Alizarin Red staining and staining quantification revealed enhanced calcific nodule staining in OM-treated cells and more pronounced staining in PCM-treated cells, due to the deposition of calcium phosphate [Ca_3_(PO_4_)_2_] in the cellular matrix (Figure 4H). These data provide evidence that pVICs can be used for *in vitro* calcification experiments.

## Discussion

Analyzing aortic valve cells is of major relevance for a better understanding of the pathology of aortic valve stenosis, but obtaining, isolating, and culturing these cells is a demanding task. Human aortic valve tissue is difficult to obtain, and the acquisition of non-stenotic control tissues is often especially challenging. In principle, living aortic valve cells can be obtained from donor hearts that are unsuitable for transplantation, as long as the aortic valve is not affected by underlying disease (29). This is the most appropriate source of control tissue, but because there are only few transplant units and a limited number of donors who would be suitable candidates, this material is difficult to obtain (30, 31). It is also possible to use explanted valves due to aortic dissection, as this is not thought to affect the aortic valve (32). However, these surgeries are typically performed on an emergency basis, with the attendant problems of informed consent and isolation procedures. The most common source of non-calcified human aortic valve tissue is patients undergoing SAVR for aortic regurgitation in the context of aortic root or ascending aortic aneurysm.

Standardization of the method of aortic valve cell isolation is crucial to ensure optimal data exchange between investigators. In a first attempt to standardize cell isolation from human and porcine aortic valve tissues, we propose a method based on previous publications with some refinements (17, 19, 23, 33, 34). We removed VECs from the AVS cusps by scraping cells from the valve tissue with the careful use of a scalpel prior to collecting and affinity-based isolation. This increased the number of VECs obtained from the isolation process compared to cotton swab removal. Moreover, we added a magnetic-activated cell sorting (MACS, Miltenyi Biotech) step with the specific endothelial marker endoglin (CD105) in order to ensure a specific VEC population (22). Although present in fibroblasts, the expression of *CD105* is typically higher in endothelial cells, thus making it an effective marker for specific isolation of VECs (35–37). This endothelial cell separation has been previously described for human vascular endothelial cells (38). To assess VEC purity, flow cytometric analysis can be used to determine the percentage of cells positive for marker genes such as PECAM1 or CDH5 (39). However, this can be challenging as the presence of residual debris, such as fibrotic material, can interfere with the flow cytometry analysis and affect the accuracy of the results. In addition, patient-specific factors, such as age, comorbidities, and medication can also affect the number of cells obtained, further complicating this analysis. For VICs, flow cytometry or MACS sorting may be challenging because there are different types of VICs in the aortic valve and no VIC-specific marker is available (40).

Based on our study, plating VICs on a collagen-coated 75 cm^2^ culture flask is more appropriate for cell culture-based experiments (17, 20). Due to the thorough sorting and lower number of VECs in general, VEC yields are significantly lower than VIC yields, and thus, porcine or human cells can be plated on a fibronectin-coated 6-well to enable cell-cell interaction (17).

The second aim of this manuscript was to compare human and porcine VECs and VICs with each other to assess whether easier-to-obtain porcine aortic valve tissue and their cells can be used for primary *in vitro* studies of human AVS pathology. We found commensurabilities but also disparities between human and porcine aortic valve cells. Under physiological conditions, hVECs and pVECs resemble endothelial cell lining the vasculature, exhibiting a typical cuboidal, endothelial cell shape with cell-junctions and expression of specific endothelial markers such as *PECAM1* and *vWF* (41). Migration characteristics differ between hVECs and pVECs. There are several potential explanations for why pVECs may not exhibit migratory capabilities comparable to those of hVECs. Disparities in extracellular matrix composition may be a factor, as well as differences in signaling pathways that control cell migration (42–44). Next generation sequencing might offer more information on genes that differ between these species. This may also provide more information on genes involved in apoptosis and why cell viability and caspase 3/7 activity were also not comparable. To test whether the differentiation of VECs into mesenchymal cells can be successfully achieved by TGFβ1, TGFβ1 + IL1β or TNFα treatment, gene expression analysis of EndMT markers was performed after 7 days (45). Because the incubation of hVECs and pVECs with TGFβ1 or TGFβ1 + IL1β did not show consistent and reliable gene expression results (Figures 1I, 3H, Supplementary Figure S2), TNFα was used for the following experiments. Current literature supports the role of TNF*α* in inducing EndMT in endothelial cells from various tissues, including the aortic valve (46, 47). In hVECs incubated with TNFα, the expression of mesenchymal cell markers such as *α-SMA, VIM*, *CDH2,* and *VCAM1* was increased, whereas endothelial markers were decreased (Figure 1I). In pVECs, endothelial markers were also downregulated, but surprisingly, so were *α-SMA* and *VIM* (Figure 3H). During this transition, activated VECs progressively lose their endothelial properties, including cell junctions, and their specific endothelial markers, and normally display increased cellular invasion and acquire a mesenchymal phenotype with improved migratory capabilities and mesenchymal marker expression (41). This result is not consistent with the current literature. Mahler and colleagues showed a successful induction of EndMT in pVECs with 100 ng/ml TNFα in 2013 (47). One possible explanation for the downregulation of *α-SMA* and *VIM* is that this concentration of TNFα may have triggered a different signaling pathway in pigs that may induce the expression of anti-inflammatory genes that can inhibit EndMT (48). Another possible reason is that the downregulation of these genes may be an early event in the process of EndMT induction. Some studies have observed that the expression of mesenchymal markers such as *ACTA2* and *VIM* may initially decrease during the early stages of EndMT, followed by a later increase (45). A time course of pVECs exposed to further concentrations of TNF*α* could be the next step. Nevertheless, next-generation sequencing of VECs that have undergone EndMT may provide further insight into the molecular distinctions between hVECs and pVECs.

hVICs and pVICs displayed the typical spindle-shaped VIC morphology with a swirling pattern that begin to grow on top of each other at confluence (23). VICs are a heterogeneous and highly plastic cell population that reside within the tissue matrix of the aortic valve (49, 50). In healthy human aortic valves, most VICs are considered to be quiescent with a fibroblast-like phenotype, which can be distinguished from VECs by positive expression of *VIM* and absence of the endothelial markers *vWF* and *PECAM1* (46, 51, 52). Upon activation, they can differentiate into activated myofibroblast-like VICs, which stain positive for *α*-SMA in addition to vimentin (49, 53). This leads to the conclusion that hVICs display a myofibroblast-like phenotype. Intriguingly, a comprehensive study identified cellular heterogeneity in aortic valve tissue and identified 234 cell clusters, representing 58 major cell types (54). This study also reported endothelial and interstitial cell heterogeneities, including an endothelial phenotype during EndMT *via* in-depth integrated multi-omics (54). Single-cell RNA sequencing and proteomic analysis of pVECs undergoing EndMT could provide further insight into the potential genetic differences. In this regard, our findings are also consistent with the above-mentioned report, suggesting a higher degree of cellular heterogeneity in the isolated porcine cell population.

Although the isolation, cultivation, and *in vitro* experiments with aortic valve cells are challenging, much progress has been made in recent years (17, 18). Different methods for isolation, EndMT, and calcification have been established worldwide (18). However, there is currently no reproducible experimental standardization, which would be crucial to ensure optimal comparisons between laboratories and to connect all results. This manuscript aims to take the first step towards standardization of human and porcine aortic valve cell isolation. *In vitro* experiments with the aortic valve, cells are immensely important to improve our understanding of the pathomechanisms involved in the development of CAVD and will undoubtedly lead to the identification of druggable targets. Additionally, no direct and detailed comparison between human and porcine aortic valve tissue has been reported to date.

**Figure 5:**
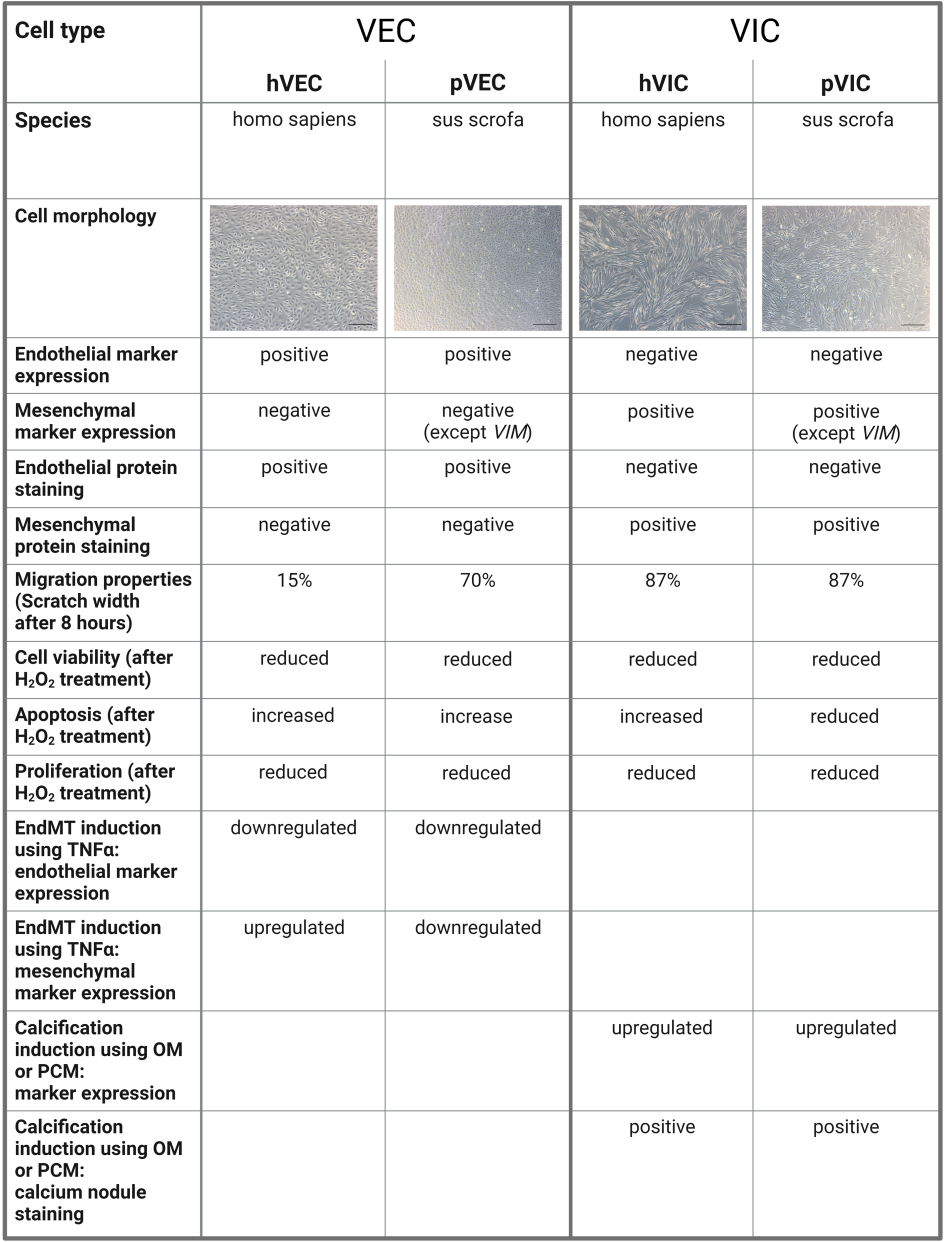
A comparison of human and porcine valvular endothelial and interstitial cells. Valvular endothelial cells (VECs), valvular interstitial cells (VICs), human VECs (hVECs), human VICs (hVICs), porcine VECs (pVECs), porcine VICs (pVICs), endothelial-to-mesenchymal transition (EndMT), tumor necrosis factor alpha (TNF*α*), osteogenic medium (OM), pro-calcifying medium (PCM).

Our study has some limitations, namely that the patient samples were all collected at the University of Bonn, Germany, and thus the patient-derived data presented are limited to the Caucasian race. Therefore, no information on race-dependent differences and heterogeneities in isolated cells from AV tissues of AVS patients undergoing SAVR could not be derived. Although the characteristic gene expression levels of VIC and VEC markers are similar in patient-derived valve cells, there are some differences in cells of porcine origin, as confirmed by RT-qPCR. Due to the technological limitations of valve cell isolations, it is still very challenging to fully confirm that valve cell populations are pure and free of other contaminants in the isolates. In cardiovascular research, a large number of studies have reported dynamic alterations in the gene expression during different cardiovascular conditions, leading to an interest in single-cell resolution investigations (55–57). Regarding patients with AVS, only some patients display impaired heart function, as evidenced by a reduction in LVEF (left ventricular ejection fraction), indicating the severity of the pathological changes of the failing heart, while other patients remain without LVEF impairment, demonstrating the heterogeneity of pathological changes during AVS (8, 13). To enumerate whether human and porcine cells are comparable in terms of cellular and other biological properties of valvular cells, we have summarized our findings in Figure 5. Such limitations can be accounted for in the case of individual experiments in specific laboratory settings in cardiovascular research (8, 13, 58).

## Conclusion

In conclusion, our findings offer new avenues for alternative cellular model systems that can be used in a wide range of biomedical research. We demonstrated for the first time in detail, that (1) patient-derived AV cells, namely, VICs and VECs, isolated from AV tissue explants of AVS patients can be isolated as a specific population. These cells express typical endothelial- and interstitial cell markers and are morphologically comparable; (2) patient-derived cells can be maintained in culture for a long time (passages, 5–6) and have the potential to be used in downstream experiments; (3) functional analysis demonstrated that isolated cells retain endothelial- and interstitial cell properties/function (e.g., in VEC: migration, cell viability, proliferation, EndMT induction; in VIC: *in vitro* calcification to facilitate osteoblastic differentiation after induction with OM or PCM); (4) a comparative and reproducible analysis of human vs. porcine cells revealed that the cell types are comparable, although some cellular heterogeneity in VECs of porcine origin (pVECs) exist.

In summary, our study has revealed an easy-to-obtain and financially viable alternative cellular model system that can be used in cardiovascular research. Our findings can also help to circumvent the limitations of accessibility and challenges in obtaining human AV tissues and provide a viable solution for lab-ready cellular models in aortic valve research.

## Data Availability

The original contributions presented in the study are included in the article/**Supplementary Material**, further inquiries can be directed to the corresponding author/s.

## Supplementary material

Supplementary Figure S1 *Human aortic valve cusp surface*. (a) human cusp surface before and after VEC removal by scalpel (VEC isolation step 2). The surface showed a roughening after scratch procedure.

Supplementary Figure S2 Expression of endothelial-to-mesenchymal (EndMT) markers after valve endothelial cell (VEC) induction with transforming growth factor beta 1 (TGFβ1) and TGFβ1 combined with interleukin beta 1 (IL1β). (a) hVECs were incubated with 1-10 ng/ml transforming growth factor beta 1 (TGFβ1) for 7 days. qPCR analysis revealed a downregulation of von Willebrand factor (*vWF*) in each condition and remained unchanged or showed upregulation of platelet adhesion molecule 1 (*PECAM1*) and cadherin 5 (*CDH5*). Snail Family Transcriptional Repressor 2 (*SNAI2*) remained unchanged and alpha smooth muscle actin (α-SMA), and vimentin (*VIM*) are partly upregulated. (b) hVECs treated with 5 ng/ml TGFβ1 in combination with 1 ng/ml interleukin 1 beta (IL1β). Gene expression analysis showed a significant reduction of *vWF* and nitric oxide synthase 3 (*NOS3*), whereas *SNAI2* and vascular cell adhesion molecule 1 (*VCAM1*) were upregulated. *PECAM1*, *α-SMA*, and *CDH2* did not show differences in gene expression. (c) pVECs were treated with 5 ng/ml TGFβ1 + 1 ng/ml IL1β for 7 days. Marker expression analysis revealed a significant reduction of all markers (vWF, PECAM1, *α-SMA*, *VIM*, *VCAM1*). (a) n=1 donor with technical replicates, (b, c) n=3 donors with technical replicates indicated by one color per donor, *P < 0.05, **P < 0.01, ***P < 0.001, ****P < 0.0001, analyzed by Student t-test, 2-tailed, unpaired.
