# Supplementary figures and images for: Human and porcine aortic valve endothelial and interstitial cell isolation and characterization
Source: Front Cardiovasc Med. 2023 Jun 20;10:1151028. doi: 10.3389/fcvm.2023.1151028 (PMC10318150; doi:10.3389/fcvm.2023.1151028)

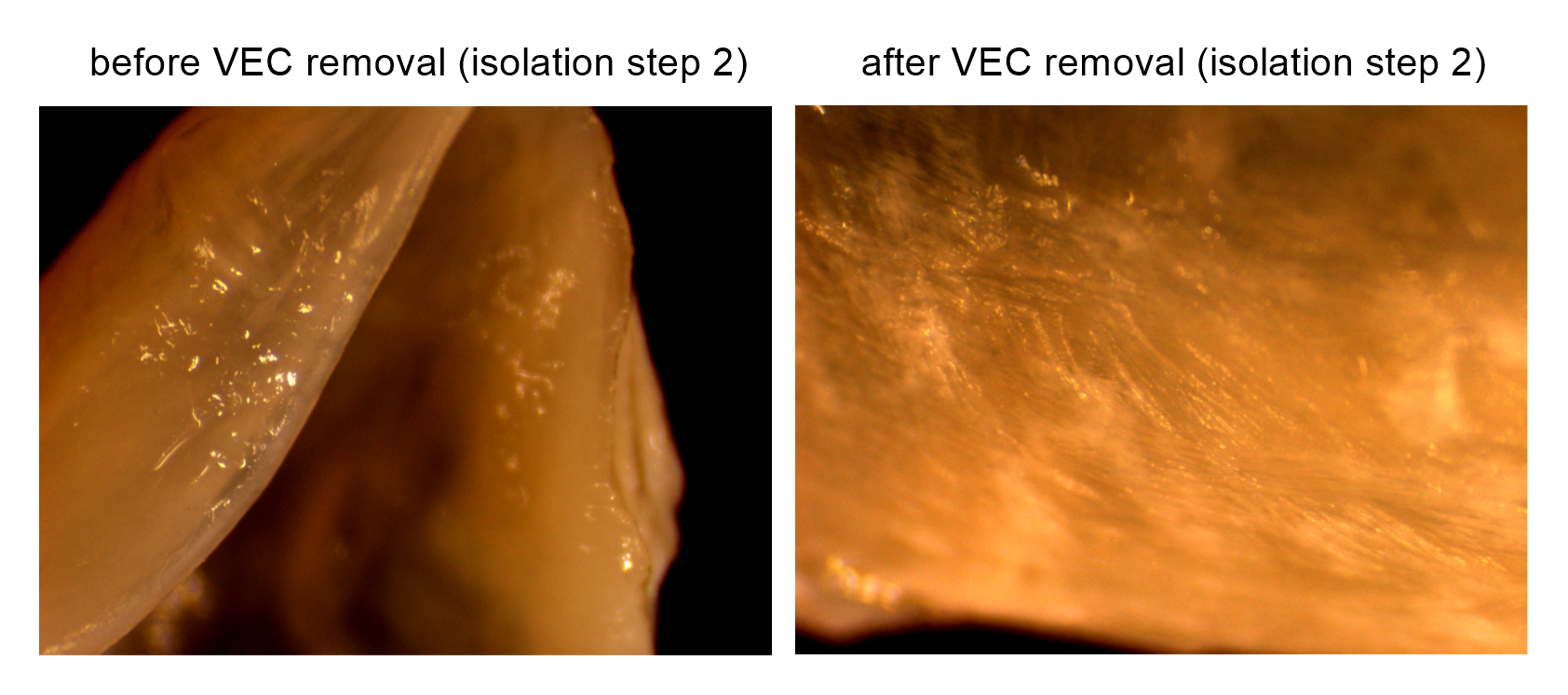

Supplement: Supplementary Figure S1 — Human aortic valve cusp surface. (a) human cusp surface before and after VEC removal by scalpel (VEC isolation step 2). The surface showed a roughening after scratch procedure. [file Image1.tif]

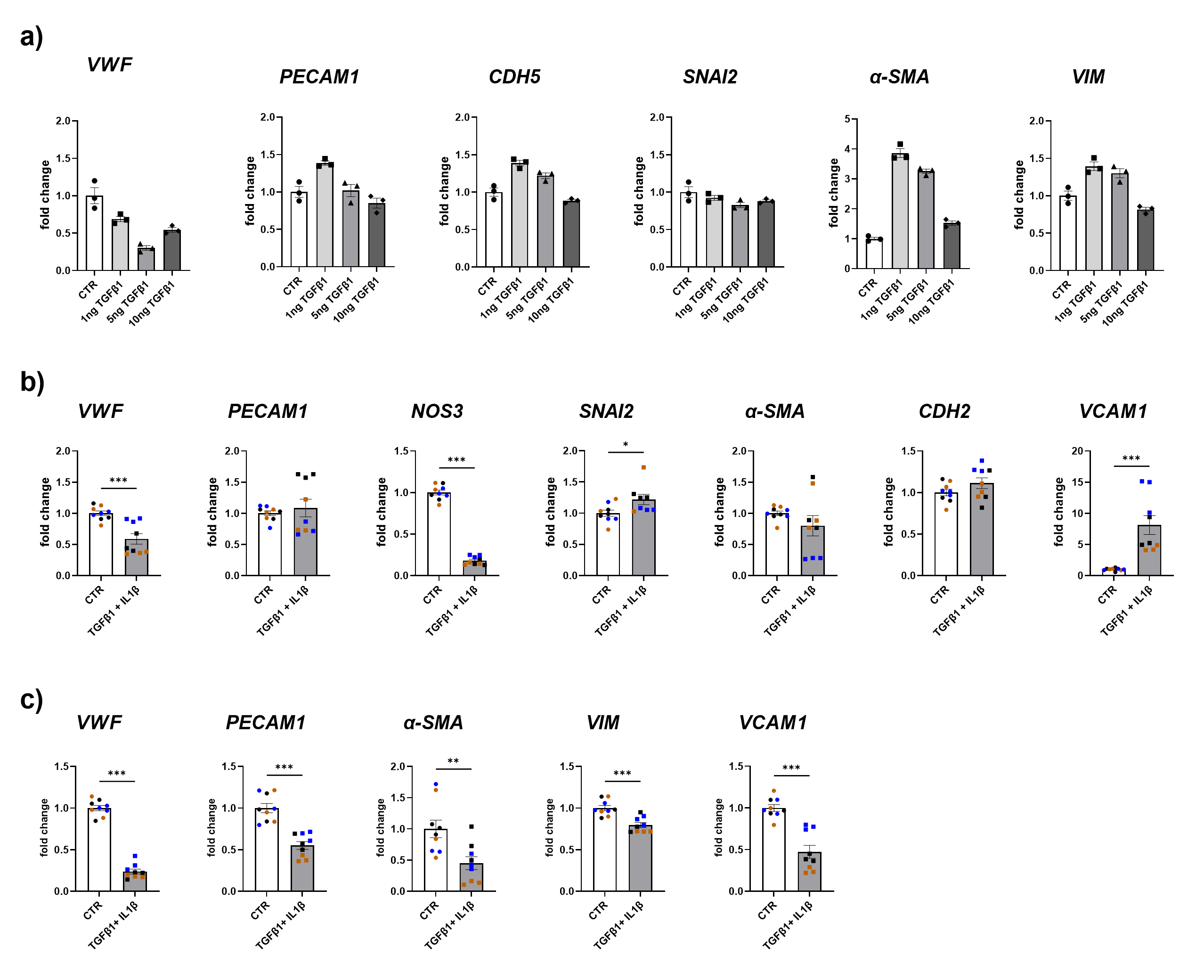

Supplement: Supplementary Figure S2 — Expression of endothelial-to-mesenchymal (EndMT) markers after valve endothelial cell (VEC) induction with transforming growth factor beta 1 (TGFβ1) and TGFβ1 combined with interleukin beta 1 (IL1β). (a) hVECs were incubated with 1-10 ng/ml transforming growth factor beta 1 (TGFβ1) for 7 days. qPCR analysis revealed a downregulation of von Willebrand factor (vWF) in each condition and remained unchanged or showed upregulation of platelet adhesion molecule 1 (PECAM1) and cadherin 5 (CDH5). Snail Family Transcriptional Repressor 2 (SNAI2) remained unchanged and alpha smooth muscle actin (α-SMA), and vimentin (VIM) are partly upregulated. (b) hVECs treated with 5 ng/ml TGFβ1 in combination with 1 ng/ml interleukin 1 beta (IL1β). Gene expression analysis showed a significant reduction of vWF and nitric oxide synthase 3 (NOS3), whereas SNAI2 and vascular cell adhesion molecule 1 (VCAM1) were upregulated. PECAM1, α-SMA, and CDH2 did not show differences in gene expression. (c) pVECs were treated with 5 ng/ml TGFβ1 + 1 ng/ml IL1β for 7 days. Marker expression analysis revealed a significant reduction of all markers (vWF, PECAM1, α-SMA, VIM, VCAM1). (a) n=1 donor with technical replicates, (b, c) n=3 donors with technical replicates indicated by one color per donor, *P < 0.05, **P < 0.01, ***P < 0.001, ****P < 0.0001, analyzed by Student t-test, 2-tailed, unpaired. [file Image2.tif]
